# Supplementary material for: Investigating the influence of physiologically relevant hydrostatic pressure on CHO cell batch culture
Source: Sci Rep. 2021 Jan 8;11:162. doi: 10.1038/s41598-020-80576-8 (PMC7794228; doi:10.1038/s41598-020-80576-8)
Supplement: Supplementary file 1 — Supplementary Information 1. [file 41598_2020_80576_MOESM1_ESM.zip › S1/FLEX2/13Jan 30mmHg SS1 is C/Jan 8 SS1.pdf]

Data File C:\Users\P...emStation\1\Data\TK_CHO_IgG 2019-01-14 15-49-10\002-4-ML Jan8 SS1.D
Sample Name: ML Jan8 SS1

===

```
Acq. Operator   : SYSTEM                         Seq. Line :   2
Sample Operator : SYSTEM
Acq. Instrument : HPLC-0039                       Location :   4
Injection Date  : 1/14/2019 3:54:55 PM                 Inj :   1
                                               Inj Volume : 100.000 µl
Method          : C:\Users\Public\Documents\ChemStation\1\Data\TK_CHO_IgG 2019-01-14 15-49-10
                  \CHO_IgG_LC.M (Sequence Method)
Last changed    : 12/4/2018 1:38:41 PM by SYSTEM
                  (modified after loading) (Current integration events modified)
```

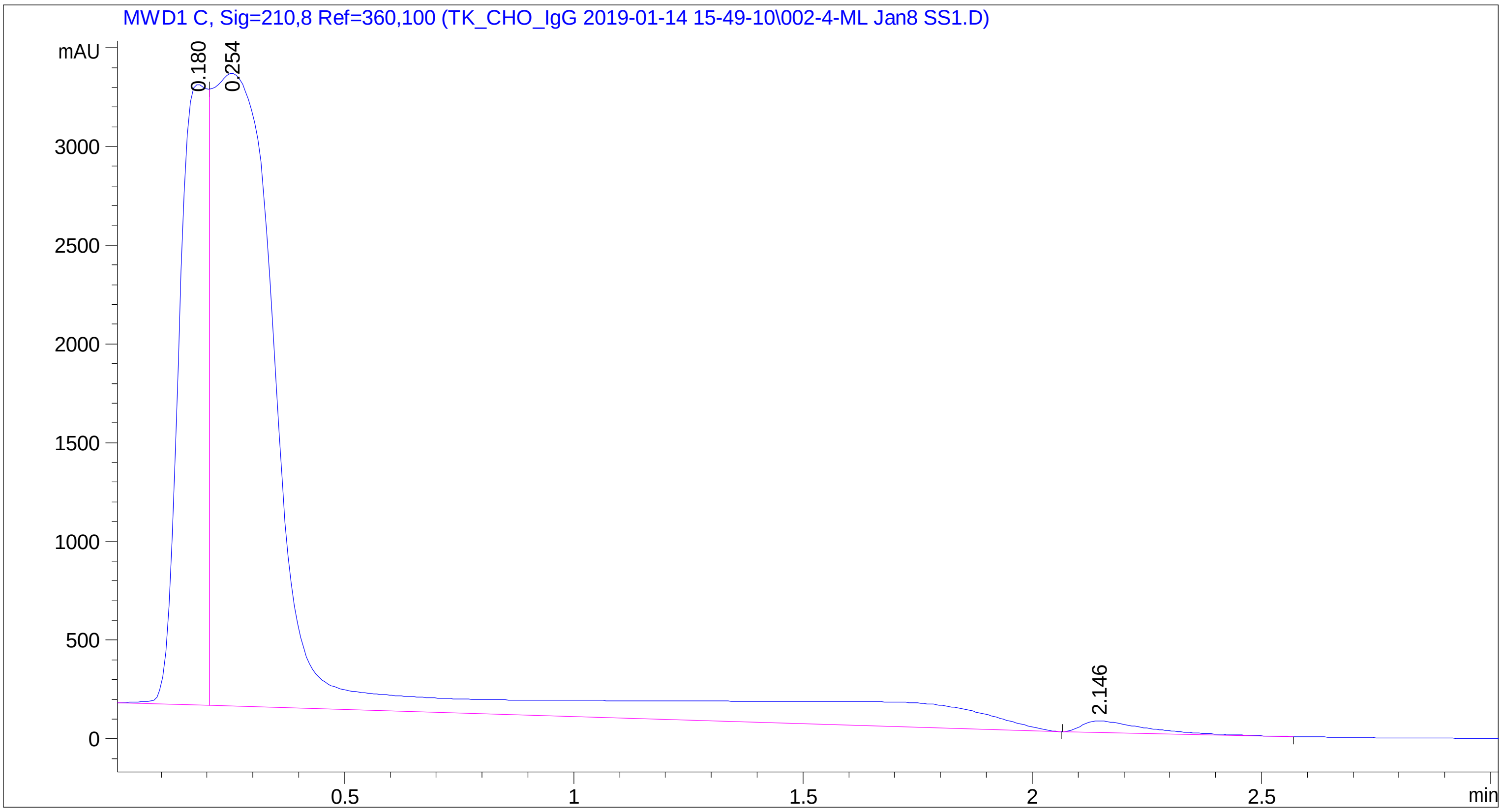

===

Area Percent Report

===

```
Sorted By             :      Signal
Multiplier            :      1.0000
Dilution              :      1.0000
Use Multiplier & Dilution Factor with ISTDs
```

Signal 1: MWD1 C, Sig=210,8 Ref=360,100

Peak #	RetTime [min]	Type	Width [min]	Area [mAU*s]	Height [mAU]	Area %
1	0.180	BV	0.0677	1.33779e4	3143.34644	26.0119
2	0.254	VB	0.1705	3.75001e4	3204.03198	72.9152
3	2.146	BBA	0.1355	551.77435	58.78227	1.0729

Totals : 5.14298e4 6406.16069

===

*** End of Report ***
